# Multisensor decentralized nonlinear fusion using adaptive cubature information filter

**DOI:** 10.1371/journal.pone.0241517

**Published:** 2020-11-05

**Authors:** Binglei Guan, Xianfeng Tang

**Affiliations:** 1 Logistics Engineering College, Shanghai Maritime University, Shanghai, China; 2 School of Electronic & Information Engineering, Ningbo University of Technology, Ningbo, China; 3 Information Technology Center, Zhejiang University, Hangzhou, China; University of Bradford, UNITED KINGDOM

## Abstract

In nonlinear multisensor system, abrupt state changes and unknown variance of measurement noise are very common, which challenges the majority of the previously developed models for precisely known multisensor fusion techniques. In terms of this issue, an adaptive cubature information filter (CIF) is proposed by embedding strong tracking filter (STF) and variational Bayesian (VB) method, and it is extended to multi-sensor fusion under the decentralized fusion framework with feedback. Specifically, the new algorithms use an equivalent description of STF, which avoid the problem of solving Jacobian matrix during determining strong trace fading factor and solve the interdependent problem of combination of STF and VB. Meanwhile, A simple and efficient method for evaluating global fading factor is developed by introducing a parameter variable named fading vector. The analysis shows that compared with the traditional information filter, this filter can effectively reduce the data transmission from the local sensor to the fusion center and decrease the computational burden of the fusion center. Therefore, it can quickly return to the normal error range and has higher estimation accuracy in response to abrupt state changes. Finally, the performance of the developed algorithms is evaluated through a target tracking problem.

## Introduction

For function and purpose of data fusion, multisensor data fusion is defined as the combination of data from a variety of sources (including sensors, or data from the network reporting) that provides inferences of a quality far superior to any single sensor or single information source [1]. Multisensor fusion techniques have long been a fascinating focus of research attracting constant attention from a variety of practical applications. These include mobile robot navigation, surveillance, air-traffic control and intelligent vehicle operations [2–5].

It is worth noting that the models of practical systems are usually nonlinear. Therefore, it is basic and important to design high performance nonlinear filter before fusion estimation. A number of suboptimal algorithms have been developed to solve the nonlinear filtering problem such as extended Kalman filter (EKF) [2], unscented Kalman filter (UKF) [6], particles filter (PF) [3], and Minimum entropy filter (MEF) [7,8]. Recently, a relatively new nonlinear filtering algorithm named cubature Kalman filter (CKF) is proposed in [9]. As a Gaussian approximation of Bayesian filter, CKF shows better filtering estimation capability than existing Gaussian filters. On the other hand, Cubature Information Filter (CIF), as the information form of CKF, is also used to make up for the deficiencies of CKF algorithm [10–12].

A limitation in aforementioned methods is that the models of system must be exact and known. However, in many nonlinear filtering or fusion problems considered in practice, the models of system are usually uncertain. These uncertainties include the sudden change of state and unknown variance of measurement noise. Strong tracking filter (STF) is a powerful tool to cope with the first case [13]. For the second case, variational Bayesian method is a good choice [14]. Naturally, a novel approach is proposed by combining the STF with the VB method [15].

Unfortunately, this method is only suitable for single sensor (i.e., local sensor). Moreover, an interdependent problem of some parameters arises in iterative calculation. The motivation of this paper is to investigate decentralized fusion problem of nonlinear multisensor system with modeling uncertainty. The key is to solve interdependent problem of combination of STF and VB, and derive efficient algorithm for computing global fading factor.

## Brief review of cubature information filter

In this section, the information form of CKF is briefly reviewed. The nonlinear filtering problems with additive process and measurement noise can be modeled using some discrete-time difference equations

x(k+1)=f(x(k))+w(k) (1)

z(k)=h(x(k))+v(k) (2)

where ***x***(*k*) ∈ ℜ^*n*×1^ and ***z***(*k*) ∈ ℜ^*m*×1^ are the state of the system and the measurement at time step*k*, respectively; ***f***(⋅) and ***h***(⋅)are some known nonlinear functions; **w**(*k*) ∈ ℜ^*n*×1^ and ***v***(*k*) ∈ ℜ^*m*×1^ are the uncorrelated zero-mean Gaussian white noises with covariances

$$\{\begin{array}{l} E[w(k)w^{T}(k)]=Q(k)\\ E[v(k)v^{T}(k)]=R(k) \end{array}$$

where ***R***(*k*) is an unknown diagonal covariance matrix.

***x***(0) is the initial target state with mean ***x***_0_ and variance ***P***_0_, and independent of ***w***(*k*) and ***v***(*k*).

The cubature information filter (CIF) is a modified version of CKF. In CIF, the information states and the inverse of the information matrix are the main two parameters that should be deal with. The details of CIF can be summarized as follows [16–18]:

### Time update

The predicted covariance and the predicted state have equivalent information forms [16–18]:

$$\{\begin{array}{l} \hat{y}(k(k-1)=Y(k)k-1)\hat{x}(k|k-1)\\ Y(k(k-1)P^{-1}(k)k-1) \end{array}$$ (3)

where

$$\hat{x}(k|k-1)=\frac{1}{2n}\overset{2n}{\underset{j=1}{\sum }}x_{j}^{*}(k|k-1)$$ (4)

$$\begin{array}{ll} P(k|k-1) & =\frac{1}{2n}\sum_{j=1}^{2n}[x_{j}^{*}(k|k-1)-\hat{x}(k|k-1)]{\left[x_{j}^{*}(k|k-1)-\hat{x}(k|k-1)\right]}^{T}+Q(k-1)\\ & ≔\overset{˜}{P}(k|k-1)+Q(k-1) \end{array}$$ (5)

$$x_{j}^{*}(k|k-1)=f(\hat{x}(k-1|k-1)+S(k-1|k-1)\xi_{j})$$ (6)

where ***S***(*k* − 1|*k* − 1) is the square root factor of ***P***(*k* − 1|*k* − 1). Point set {***ξ***_*i*_}is defined in [9].

### Measurement update

With respect to ***ŷ*** (*k*|*k* − 1) and ***Y***(*k*|*k* − 1), their updated expressions are given by

$$\{\begin{array}{l} \hat{y}(k|k)=\hat{y}(k|k-1)+i(k)\\ Y(k|k)=Y(k|k-1)+I(k) \end{array}$$ (7)

Here, the contribution of information state ***i***(*k*) and the corresponding information matrix ***I***(*k*) are given in [16–18]

$$\begin{array}{ll} i(k) & =Y(k|K-1)P_{xz}(k|k-1)R^{-1}(k)[z(k)-\hat{z}(k|k-1)+P_{xz}^{T}(k|k-1)\hat{y}(k)k-1)]\\ & ≔H^{T}(k)R^{-1}(k)[\gamma (k|k-1)+P_{xz}^{T}(k|k-1)\hat{y}(k|k-1)] \end{array}$$ (8)

$$\begin{array}{ll} I(k) & =Y(k|K-1)P_{xz}(k|k-1)R^{-1}(k)[Y(k|k-1)P_{xz}{\left(k|k-1)\right]}^{T}\\ & ≔H^{T}(k)R^{-1}(k)H(k) \end{array}$$ (9)

where

$$\{\begin{array}{l} H(k)=P_{xz}^{T}(k|k-1)Y^{T}(k|k-1)\\ \gamma (k)=z(k)-\hat{z}(k|k-1) \end{array}$$ (10)

is ***H***(*k*) is pseudo measurement matrix. ***γ***(*k*) is measurement innovation. The predicted measurement $\hat{z}(k|k-1)$ and cross-covariance ***P***_xz_(*k*|*k* − 1) are given in [16–18]

$$\{\begin{array}{l} z_{j}^{*}(k|k-1)=h(\hat{x}(k|k-1)+S(k|k-1)\xi_{j})\\ \hat{z}(k|k-1)=\frac{1}{2n}\sum_{j=1}^{2n}z_{j}^{*}(k|k-1)\\ P_{xz}(k|k-1)=\frac{1}{2n}\sum_{j=0}^{2n}[x_{j}^{*}(k|k-1)-{\hat{x}}_{i}(k|k-1)]{\left[z_{j}^{*}(k|k-1)-{\hat{z}}_{i}(k|k-1)\right]}^{T} \end{array}$$ (11)

where ***S***(*k*|*k* − 1) is the square root factor of ***P***(*k*|*k* − 1).

Finally, the state vector and the covariance matrix can be therefore obtained by

$$\{\begin{array}{l} \hat{x}(k|k)=Y(k|k)\backslash \hat{y}(k|k)\\ P(k|k)=Y(k|k)\backslash E_{n} \end{array}$$ (12)

where ***E***_*n*_ is the *n* × *n* identity matrix.

## Adaptive CIF using strong tracking filter and variational bayesian

However, CIF has bad performance in practical system with modeling uncertainty. In this article, we consider two cases of uncertainty. One is the sudden change of state; the other is unknown variance of measurement noise. In view of those, we introduce strong tracking filter technology and variational Bayesian technology to improve CIF, and propose an adaptive CIF (ACIF-STF-VB).

### Strong tracking filter

The strong tracking filter (STF) based on the orthogonal theory can reduce adaptively estimate bias and thus has ability to track abrupt changes in nonlinear systems [13]. Therefore, for the CIF, a modified state prediction error covariance with the fading factor *λ*(*k*) is given in [16,17]

$$P(k|k-1)=\lambda (k)\tilde{P}(k|k-1)+Q(k-1)$$ (13)

The time-varying suboptimal fading factor *λ*(*k*) can be calculated as follows:

$$\lambda (k)=\left\{ \begin{array}{cc} c(k), & c(k)>1\\ 1, & c(k)\leq 1 \end{array}\right.$$ (14)

where, *c*(*k*) = *tr*[***N***(*k*)]/*tr*[***M***(*k*)], and *N*(k) and *M*(k) is given in [16,17]

$$\{\begin{array}{l} N(k)=V_{0}(k)-\tau R(k)-H(k)Q(k-1)H^{T}(k)\\ M(k)=H(k)\tilde{P}(k|k-1)H^{T}(k) \end{array}$$ (15)

where,

$$V_{0}(k)=\{\begin{array}{cc} \gamma (1)\gamma^{T}(1), & k=1\\ \frac{\left[\varphi V_{0}(k-1)+\gamma (k)\gamma^{T}(k)\right]}{1+\varphi }, & k>1 \end{array}$$ (16)

where, the forgetting factor *φ* should be 0 < *φ* ≤ 1. The softening factor *τ* ≥ 1plays a role of smoothing the state estimation. It is worth noting that some Jacobian matrixes are not computed.

In order to guarantee the normal execution of the algorithm, we need to compute pseudo measurement matrix ***H***(*k*) and innovation ***γ***(*k*). Those parameters can be evaluated as follows:

1. Denote $P_{ori}(k|k-1)=\tilde{P}(k|k-1)+Q(k-1)$ is the original prediction error covariance.
2. Then, substituting it into Eq (11) yields ${\hat{z}}_{ori}=(k|k-1)$ and ***P***_*ori*,*xz*_(*k*|*k* − 1).
3. Finally, calculate ***H***(*k*) and ***γ***(*k*) by Eq (10).

### Variational bayesian approximation

Clearly, the fading factor *λ*(*k*) cannot be determined if the variance ***R***(*k*) is unknown. But VB can help to estimate ***R***(*k*) online. VB approximates the optimal posteriori distribution of state and measurement noise by a free form distribution of the factorization. Based upon VB, estimation formula of variance ***R***(*k*) are suggested in [14,15,18]:

1. Evaluate parameters predict of variance ***R***(*k*)
  $$\{\begin{array}{l} \alpha (k|k-1)=\rho •\alpha (k-1)\\ \beta (k|k-1)=\rho •\beta (k-1) \end{array}$$ (17)
  where “•” is the point operation in Matlab. ρ,α(k), β(k) are all m-dimensional column vectors and *ρ*_*i*_ ⊂ (0,1)(i = 1,⋯,m)
2. Iteration initialization, set t = 0 and the number of iterations be N_1_. In practical application, N_1_ is usually no more than 3., and
  $$\{\begin{array}{l} \alpha (k)={\left[1/2,\cdots 1/2\right]}^{T}+\alpha (k|k-1)\\ \beta^{0}(k)=\beta (k|k-1) \end{array}$$ (18)
3. Compute the covariance of measurement noise
  $${\hat{R}}^{t}(k)=diag(\beta^{t}(k)•/\alpha (k))$$ (19)
  where operation diag(*) means that diagonal entries of matrix ‘*’ form a column vector.
4. Estimate the updated state ${\hat{x}}^{t+1}(k|k)$ and its error covariance ***P***^*t*+1^(*k*|*k*) by Eqs (3)–(12).
5. Update parameter ***β***^*t*+1^(*k*)
  $$\beta^{t+1}(k)=\beta (k|k-1)+{\left[z(k)-H(k){\hat{x}}^{t+1}(k|k)\right]}^{•2}/2+diag\{H(k)P^{t+1}(k|k)H^{T}(k)\}/2$$ (20)
6. t = t+1, repeat step 2)-6) until t = *N*_1_.
7. Complete the iteration, and
  $$\{\begin{array}{l} \beta (k)=\beta^{N_{1}}(k)\\ \hat{R}(k)={\hat{R}}^{N_{1}}(k) \end{array}$$ (21)

### ACIF-STF-VB

In this subsection, a novel adaptive filter is presented by embedding the fading factor and Variational Bayesian estimation of ***R***(*k*), which is called ACIF-STF-VB. The procedure of the ACIF-STF-VB is described as follows:

1. Evaluate predicted state $\hat{x}(k|k-1)$ by Eq (4).
2. Estimate ${\hat{R}}^{t}(k)$ according to Eqs (17)–(19).
3. Calculate ***H***(*k*) and ***γ***(*k*) in terms of step a)-c) in subsection of strong tracking filter.
4. Compute *λ*(*k*), ***Y***(*k*|*k* − 1) and $\hat{y}(k|k-1)$ by Eqs (3), (13)–(16).
5. Iteratively estimate ${\hat{x}}^{t}(k|k)$ and ***P***^*t*^(*k*|*k*) by using Eqs (7)–(12).
6. If *t* < *N*_1_, update parameter ***β***^*t*+1^(*k*) by Eq (20), let *t* = *t* + 1 and go back to ii); else go to vii).
7. Iteration completed, and
  $$\{\begin{array}{l} \beta (k)=\beta^{N_{1}}(k)\\ \hat{R}(k)={\hat{R}}^{N_{1}}(k) \end{array},\{\begin{array}{l} \hat{x}(k|k)={\hat{x}}^{N_{1}}(k|k)\\ P(k|k)=P^{N_{1}}(k|k) \end{array}$$

Obviously, estimating variance ***R***(*k*) and determining the fading factor *λ*(*k*) are interdependent in ACIF-STF-VB. There are many differences between ACIF-STF-VB with VB-ACSTIF presented in [15]. 1) All Jacobian matrixes are avoided to calculate in ACIF-STF-VB. 2) Interdependent problem on the computation of ***H***(*k*) and *λ*(*k*) is solved by introduce original error covariance ***P***_*ori*_(*k*|*k* − 1). 3) In VB-ACSTIF, only iteration estimate ${\hat{R}}^{0}(k)$ is used to evaluate *λ*(*k*). In ACIF-STF-VB, both ${\hat{R}}^{t}(k)$ and *λ*(*k*) are corrected in each iteration. In a word, ACIF-STF-VB has better performance than VB-ACSTIF.

## ACIF-STF-VB in multisensor decentralized fusion

**Fig 1:**
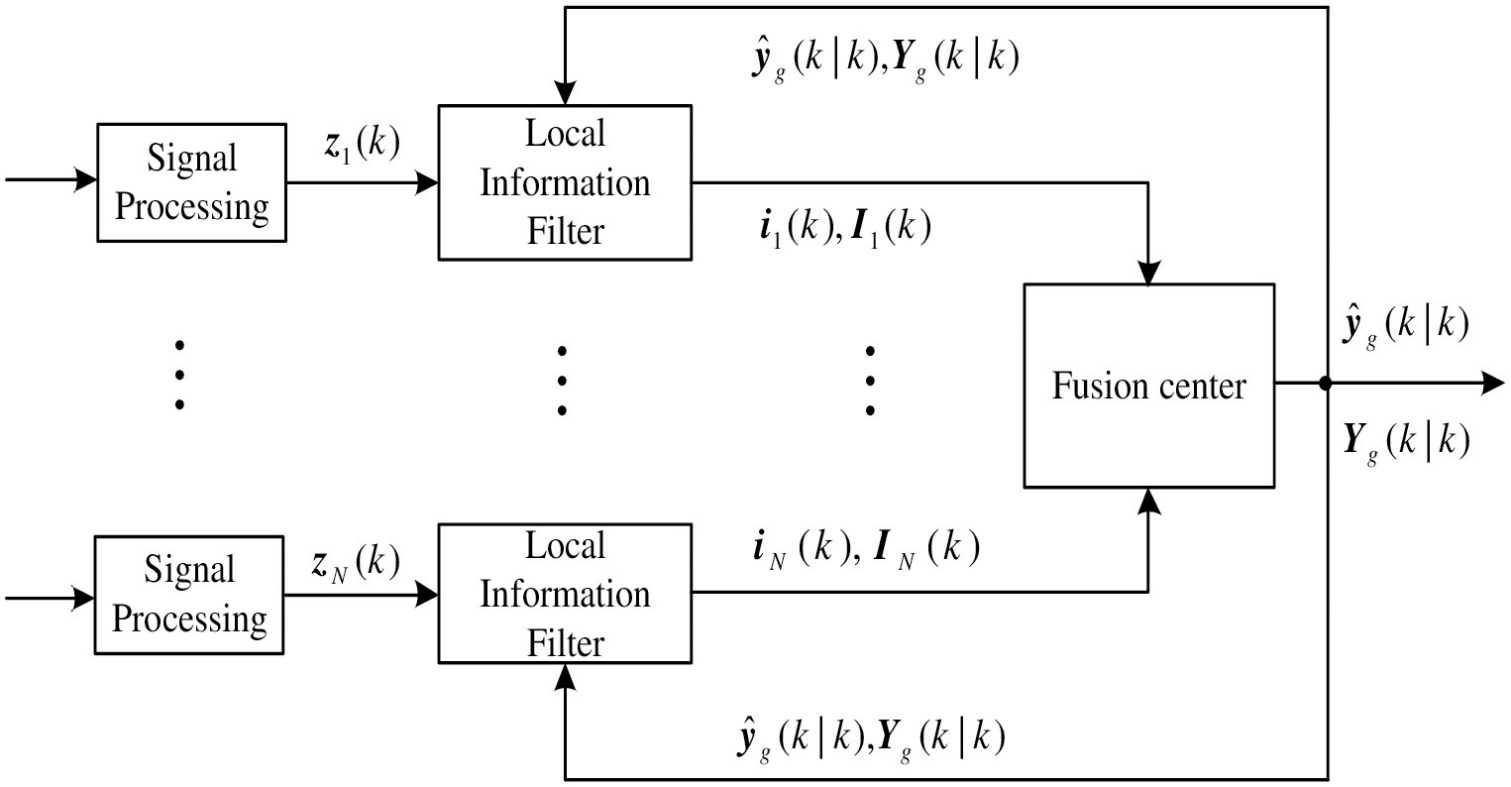
Information filter for decentralized fusion.

In this section, we specifically consider a decentralized fusion framework with feedback. As shown in Fig 1, in this configuration, each local sensor has its own information processor. Compared with processing characteristics of Kalman filters, the decentralized fusion structure is easier to fuse information from multiple sensors, simply by adding information contributions to the information vector and associated matrix.

Suppose the different sensors used for state estimation be given by

$$z_{s}(k)=h_{s}(x(k))+v_{s}(k),s=1,2,\cdots ,N$$ (22)

where, ***z***_*s*_(*k*) ∈ ***R***^*m*×1^ is a measurement vector from the sensor s (*s* = 1,2,⋯, *N*); ***h***_*s*_(⋅) is the corresponding nonlinear functions; ***v***_*s*_(*k*) ∈ ***R***^*m*×1^ is the zero-mean Gaussian white noises with diagonal covariances ***R****_s_*(*k*).

Suppose that information contributions (***i***_*s*_(k) and ***I***_*s*_(k)) of each sensor are available at time *k*. Then, the updated information vector and information matrix in fusion center can be obtained as follows

$${\hat{y}}_{g}(k|k)={\hat{y}}_{g}(k|k-1)+\overset{N}{\underset{s=1}{\sum }}i_{s}(k)$$ (23)

$$Y_{g}(k|k)=Y_{g}(k|k-1)+\overset{N}{\underset{s=1}{\sum }}I_{s}(k)$$ (24)

where

$$\{\begin{array}{l} {\hat{y}}_{g}(k|k-1)=Y_{g}(k|k-1){\hat{x}}_{g}(k|k-1)\\ \begin{array}{l} Y_{g}(k|k-1)=P_{{}_{g}}^{-1}(k|k-1)\\ P_{g}(k|k-1)=\lambda_{g}(k){\tilde{P}}_{g}(k|k-1)+Q(k-1) \end{array} \end{array}$$ (25)

Therefore, the key for fusion estimation is to compute global fading factor*λ*_*g*_(*k*). Clearly, *λ*_*g*_(*k*) cannot be simply formulated using local fading factor *λ*_*s*_(*k*). In order to reveal the relationship between two fading factors, we will rewrite the calculation process of *λ*_*s*_(*k*). Define a five-dimensional parameter vector (This parameter is named as fading vector) of the sensor s as follows:

$$p_{s}(k)={\left[p_{s,1}(k),p_{s,2}(k),p_{s,3}(k),p_{s,4}(k),p_{s,5}(k)\right]}^{T}$$ (26)

where

$$\{\begin{array}{l} p_{s,1}(k)=tr[V_{s}^{0}(k)]\\ p_{s,2}(k)=tr[R_{s}(k)]\\ p_{s,3}(k)=tr[H_{s}(k)Q(k-1)H_{s}^{T}(k)]\\ p_{s,4}(k)=tr[H_{s}(k){\tilde{P}}_{s}(k|K-1)H_{s}^{T}(k)]\\ p_{s,5}(k)=tr[\gamma_{s}(k)\gamma_{s}^{T}(k)] \end{array}$$

Combining Eq (26) with Eqs (14)–(23), it obtains

$$\lambda_{s}(k)=\{\begin{array}{cc} c_{s}(k), & c_{s}(k)>1\\ 1, & c_{s}(k)\leq 1 \end{array}$$ (27)

where, *c*_*s*_(*k*) = *q*_*s*_(*k*)/*p*_*s*,*4*_(*k*), and

$$q_{s}(k)=p_{s,1}(k)-\tau \cdot p_{s,2}(k)-p_{s,3}(k)$$ (28)

where

$$p_{s,1}(k)=\{\begin{array}{cc} p_{s,5}(1), & k=1\\ \frac{\phi \cdot p_{s,1}(k-1)+p_{s,5}(k)}{1+\phi }, & k>1 \end{array}$$ (29)

That is to say, *λ*_*s*_(*k*) can be determined by fading vector ***p***_*s*_(*k*) in terms of Eqs (27)–(29). Similarly, we can define ***p***_*g*_(*k*) as a global fading vector. Then, we can contain the following theorem 1.

**Theorem 1** For decentralized fusion using strong tracking filter, if all local fading vector ***p***_*s*_(*k*)are available at time *k*, then the global fading vector ***p***_*g*_(*k*)can be calculate in form

$$p_{g}(k)=\overset{N}{\underset{s=1}{\sum }}p_{s}(k)$$ (30)

***Proof***: Denote

$$H_{g}(k)=[\begin{array}{c} H_{1}(k)\\ \vdots \\ H_{n}(k) \end{array}],\gamma_{g}=[\begin{array}{c} \gamma_{1}(k)\\ \vdots \\ \gamma_{n}(k) \end{array}],R_{g}=[\begin{array}{ccc} {\hat{R}}_{1} & & \\ & \ddots & \\ & & {\hat{R}}_{N} \end{array}]$$

According to the definition of ***p***_*g*_(*k*), We have

$$p_{g}(k)={\left[p_{g,1}(k),p_{g,2}(k),p_{g,3}(k),p_{g,4}(k),p_{g,5}(k)\right]}^{T}$$ (31)

where,

$$\{\begin{array}{l} p_{g,1}(k)=tr[V_{g}^{0}(k)]\\ p_{g,2}(k)=tr[R_{g}(k)]\\ p_{g,3}(k)=tr[H_{g}(k)Q(k-1)H_{s}^{T}(k)]\\ p_{g,4}(k)=tr[H_{g}(k){\tilde{P}}_{g}(k|K-1)H_{g}^{T}(k)]\\ p_{g,5}(k)=tr[\gamma_{g}(k)\gamma_{g}^{T}(k)] \end{array}$$

Second component of ***p***_*g*_(*k*) is

$$p_{g,2}(k)=tr[R_{g}(k)]=\overset{N}{\underset{s=1}{\sum }}tr[R_{s}(k)]=\overset{N}{\underset{s=1}{\sum }}p_{s,2}(k)$$ (32)

Third components of ***p***_*g*_(*k*)is

$$\begin{array}{ll} p_{g,3}(k) & =tr[H_{g}(k)Q(k-1)H_{g}^{T}(k)]\\ & =\sum_{s=1}^{N}tr[H_{s}(k)Q(k-1)H_{s}^{T}(k)]\\ & =\sum_{s=1}^{N}p_{s,3}(k) \end{array}$$ (33)

Fourth component of ***p***_*g*_(*k*) is

$$\begin{array}{ll} p_{g,4}(k) & =tr[H_{g}(k){\tilde{P}}_{g}(k|k-1)H_{g}^{T}(k)]\\ & =\sum_{s=1}^{N}tr[H_{s}(k){\tilde{P}}_{g}(k|k-1)H_{s}^{T}(k)]\\ & =\sum_{s=1}^{N}tr[H_{s}(k){\tilde{P}}_{s}(k|k-1)H_{s}^{T}(k)]\\ & =\sum_{s=1}^{N}p_{s,4}(k) \end{array}$$ (34)

where ${\tilde{P}}_{g}(k|k-1)={\tilde{P}}_{s}(k|k-1)$ is used in above equation. This is because there is a feedback from fusion center to local sensor.

Fifth component of ***p***_*g*_(*k*) is

$$\begin{array}{ll} p_{g,5}(k) & =tr[\gamma_{g}(k)\gamma_{g}^{T}(k)]\\ & =\sum_{s=1}^{N}tr[\gamma_{s}(k)\gamma_{s}^{T}(k)]\\ & =\sum_{s=1}^{N}p_{s,5}(k) \end{array}$$ (35)

Finally, we discuss first component.

If *k* = 1, we have

$$\begin{array}{ll} p_{g,1}(1) & =tr[V_{g}^{0}(1)]=tr[\gamma_{g}(1)\gamma_{g}^{T}(1)]\\ & =\sum_{s=1}^{N}p_{s,5}(1)=\sum_{s=1}^{N}p_{s,1}(1) \end{array}$$ (36)

So, $p_{g,1}(k)=\sum_{s=1}^{N}p_{s}{}_{,1}(k)$ holds if *k* = 1.

If *k* = 2, we get

$$\begin{array}{ll} p_{g,1}(2) & =tr[V_{g}^{0}(2)]\\ & =tr\{\frac{\varphi V_{g}^{0}(1)+\gamma_{g}(2)\gamma_{g}^{T}(2)}{1+\varphi }\}\\ & =\frac{\varphi p_{g,1}(1)+p_{g,5}(2)}{1+\varphi }\\ & =\frac{\varphi \sum_{s=1}^{N}p_{s,1}(1)+\sum_{s=1}^{N}p_{s,5}(2)}{1+\varphi }\\ & =\sum_{s=1}^{N}\frac{\varphi p_{s,1}(1)+p_{s,5}(2)}{1+\varphi }\\ & =\sum_{s=1}^{N}p_{s,1}(2) \end{array}$$ (37)

That is to say, $p_{g,1}(k)=\sum_{s=1}^{N}p_{s}{}_{,1}(k)$ holds if *k* = 2.

Assume that *k* = *l* (*l* > 2), $p_{g,1}(l)=\sum_{s=1}^{N}p_{s}{}_{,1}(l)$, then

$$\begin{array}{l} p_{g,1}(l+1)=tr[V_{g}^{0}(l+1)]\\ =tr\{\frac{\varphi V_{g}^{0}(l)+\gamma_{g}(l+1)\gamma_{g}^{T}(l+1)}{1+\varphi }\}\\ =\frac{\varphi p_{g,1}(l)+p_{g,5}(l+1)}{1+\varphi }\\ =\frac{\varphi \sum_{s=1}^{N}p_{s,1}(l)+\sum_{s=1}^{N}p_{s,5}(l+1)}{1+\varphi }\\ =\sum_{s=1}^{N}\frac{\varphi p_{s,1}(l)+p_{s,5}(l+1)}{1+\varphi }\\ =\sum_{s=1}^{N}p_{s,1}(l+1) \end{array}$$ (38)

In a word, $p_{g,1}(k)=\sum_{s=1}^{N}p_{s}{}_{,1}(k)$ holds for arbitrarily *k*.

In summary, $p_{g}(k)=\sum_{s=1}^{N}p_{s}(k)$ always holds. So, the proof of theorem 1 is completed.

According to theorem 1 and combining with Eqs (27)–(29), it’s easy to yield

$$\lambda_{g}(k)=\{\begin{array}{cc} c_{g}(k), & c_{g}(k)>1\\ 1, & c_{g}(k)\leq 1 \end{array}$$ (39)

where, *c*_*g*_(*k*) = *q*_*g*_(*k*)/*p*_*g*,*4*_(*k*), and

$$q_{g}(k)=p_{g,1}(k)-\tau \cdot p_{g,2}(k)-p_{g,3}(k)$$ (40)

where

$$p_{g,1}(k)=\{\begin{array}{cc} p_{g,5}(1), & k=1\\ \frac{\varphi \cdot p_{g,1}(k-1)+p_{g,5}(k)}{1+\varphi }, & k>1 \end{array}$$ (41)

In this section, we develop an effective method for computing global fading factor ***p***_*g*_(k) by introducing fading vectors ***p***_*s*_(k). Obviously, the proposed method reduces the computational demands of fusion center, but also reduces the burden of communication transmission.

## Numerical examples

To test and verify the performance of ACIF-STF-VB and the decentralized fusion algorithm (called DF-ACIF-STF-VB), we consider the problem of tracking a target in two-dimensional space.

The target moves as a CV model, which is given by

$$x(k)=[\begin{array}{cccc} 1 & \Delta T & 0 & 0\\ 0 & 1 & 0 & 0\\ 0 & 0 & 1 & \Delta T\\ 0 & 0 & 0 & 1 \end{array}]x(k-1)+w(k-1)$$

where ***x***(*k*) is the state of the aircraft and $x(k)={\left[x(k),\dot{x}(k),y(k),\dot{y}(k)\right]}^{T}$. *x*(*k*) and *y*(*k*) are the coordinates for the position of the aircraft.$\dot{x}(k)$ and $\dot{y}(k)$ are the velocity in the corresponding axis. Δ*T* is the sampling time, and Δ*T* = 1s. In this section, the simulation time is 100s. ***w***(*k* − 1) is a zero-mean Gaussian noise vector with covariance ***Q***, and

$$Q=[\begin{array}{cccc} \frac{{\left(\Delta T\right)}^{3}}{3} & \frac{{\left(\Delta T\right)}^{2}}{2} & 0 & 0\\ \frac{{\left(\Delta T\right)}^{2}}{2} & \Delta T & 0 & 0\\ 0 & 0 & \frac{{\left(\Delta T\right)}^{3}}{3} & \frac{{\left(\Delta T\right)}^{2}}{2}\\ 0 & 0 & \frac{{\left(\Delta T\right)}^{2}}{2} & \Delta T \end{array}]\cdot q(k)$$

where *q*(*k*) is power spectral density and *q*(*k*) = 0.15m^2^⋅s^−3^.

Consider a nonlinear target tracking system composed of three radars and the nonlinear measurement model is expressed as

$$z_{i}(k)=[\begin{array}{c} \sqrt{{\left(x(k)-x_{s,i}\right)}^{2}+{\left(y(k)-y_{s,i}\right)}^{2}}\\ \text{arctan}(\frac{y(k)-y_{s,i}}{x(k)-x_{s,i}}) \end{array}]+v_{i}(k),i=1,2,3$$

where (*x*_*s*,*i*_, *y*_*s*,*i*_) is the position of the radar *i*. Suppose the position axis of three radars are (0m, 0m), (-2000m, 0m) and (2000m, 0m). ***v***_*i*_(*k*) is an additive zero-mean Gaussian noise vector with variance $R_{i}(k)=diag[\sigma_{r,i}^{2},\sigma_{b,i}^{2}]$. *σ*_*r*,*i*_ = 5m and *σ*_*b*,*i*_ = 0.1°.

We assume ***R***_*i*_(0) = *diag*[(81m)^2^, (0.3°)^2^] is known so that the algorithms can normally work. For the STF and VB method in examples, the corresponding parameters are *φ* = 0.95, *τ* = 3; *N*_1_ = 2, ***ρ*** = [1 − *e*^−6^, 1 − *e*^−6^]^*T*^, ***α***(0) = [1,1]^*T*^, ***β***(0) = [100,0.05]^*T*^.

Initial state and error covariance matrix are set

$$x_{0}={\left[2000\text{m},150\text{m/s},2500\text{m},200\text{m/s}\right]}^{T};p_{0}=\text{diag}[{(100\text{m)}}^{2},{(15\text{m/s)}}^{2},{(100\text{m)}}^{2},{(15\text{m/s)}}^{2}]$$

For comparison purposes, we use the square error of position (SEP(k)), square error of velocity (SEV(k)), mean square error of position (MSEP) and mean square error of velocity (MSEV) defined in [19].

### Example 1

**Fig 2:**
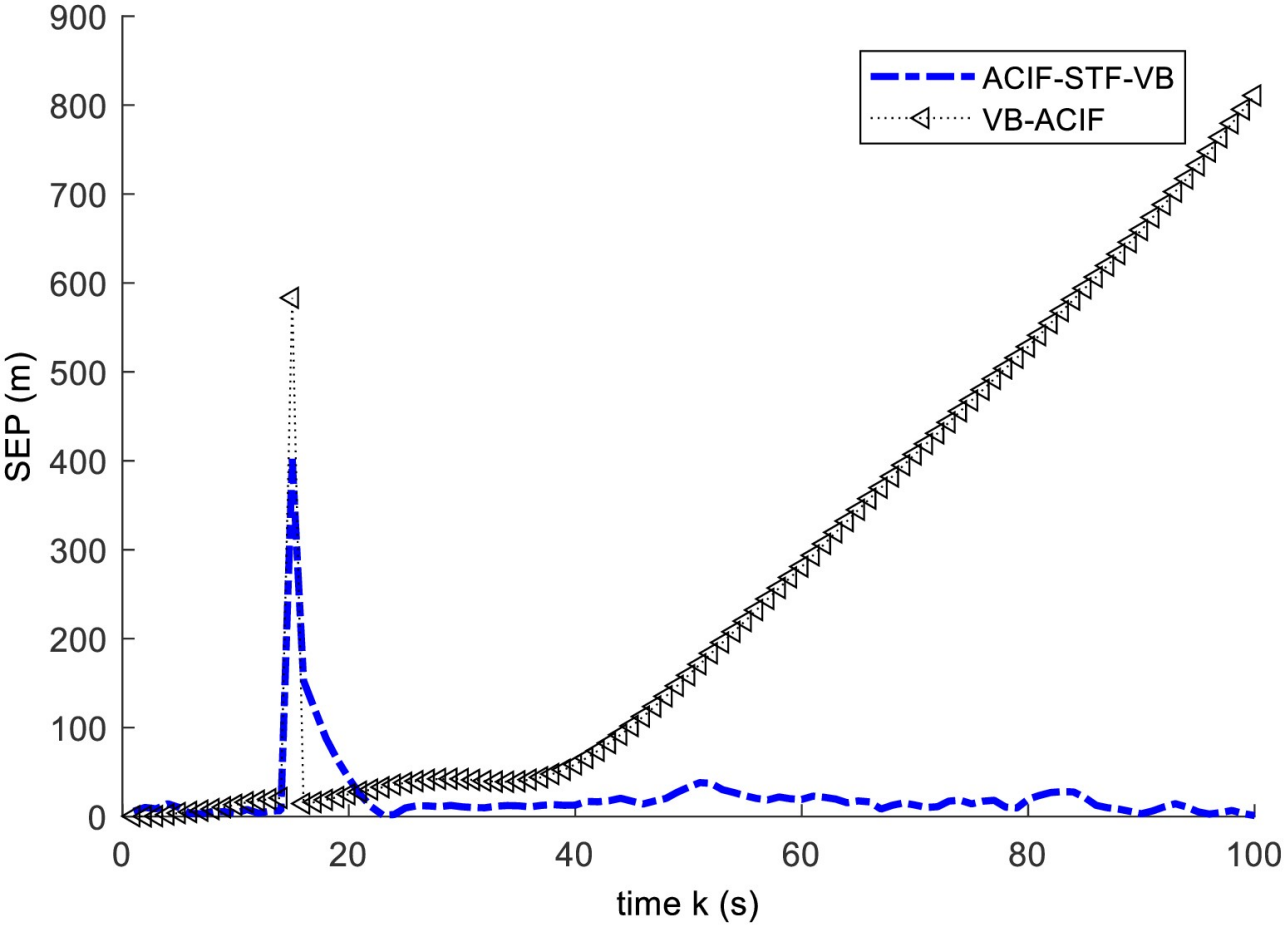
Square error curves of the position.

**Fig 3:**
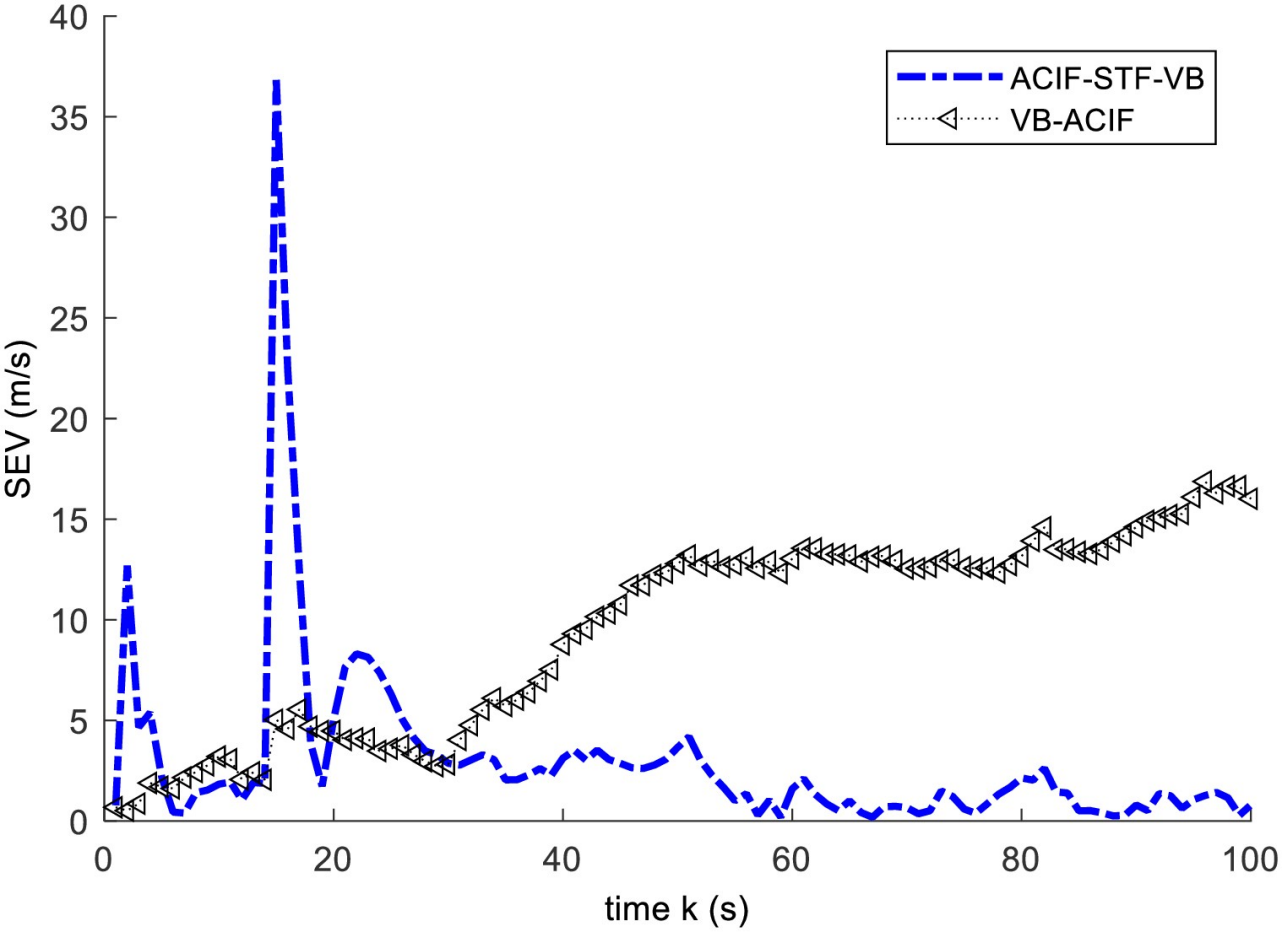
Square error curves of the velocity.

This example is used to validate the proposed ACIF-STF-VB compared with VB-ACIF in [15]. In this case, an abrupt state change happens at *k* = 15s. We set *x*(*k*) = 1.1*x*(*k*), *y*(*k*) = 1.1*y*(*k*). For simplicity, we choose the radar fixed at the origin. We compare the two algorithms by the square error of position and the square error of velocity. The simulation results are illustrated by Figs 2 and 3.

From Figs 2 and 3, the results show: 1) When the target is in normal motion within 0~15s, both of the algorithms can track the target well. 2) As an abrupt state change take place at 15s, both of the algorithms have large tracking errors simultaneously. 3) After a short time of adjustment, the ACIF-ST-VB algorithm can track the target quickly, and the tracking error curve of the response tends to converge. In contrast, the tracking error of VB-ACIF algorithm is increasing and the curve diverges gradually after the target state mutation, which means it can no longer track the target.

The mean square error of two methods is given by Table 1. The results show that ACIF-STF-VB has better accuracy than VB-ACIF. Obviously, the strong tracking fading factor improves the accuracy and stability of the algorithm.

**Table 1:** Mean square error of two algorithms.

Algorithm	ACIF-STF-VB	VB-ACIF
MSEP (m)	21.8446	266.3492
MSEV (m/s)	2.9034	9.4795

### Example 2

**Fig 4:**
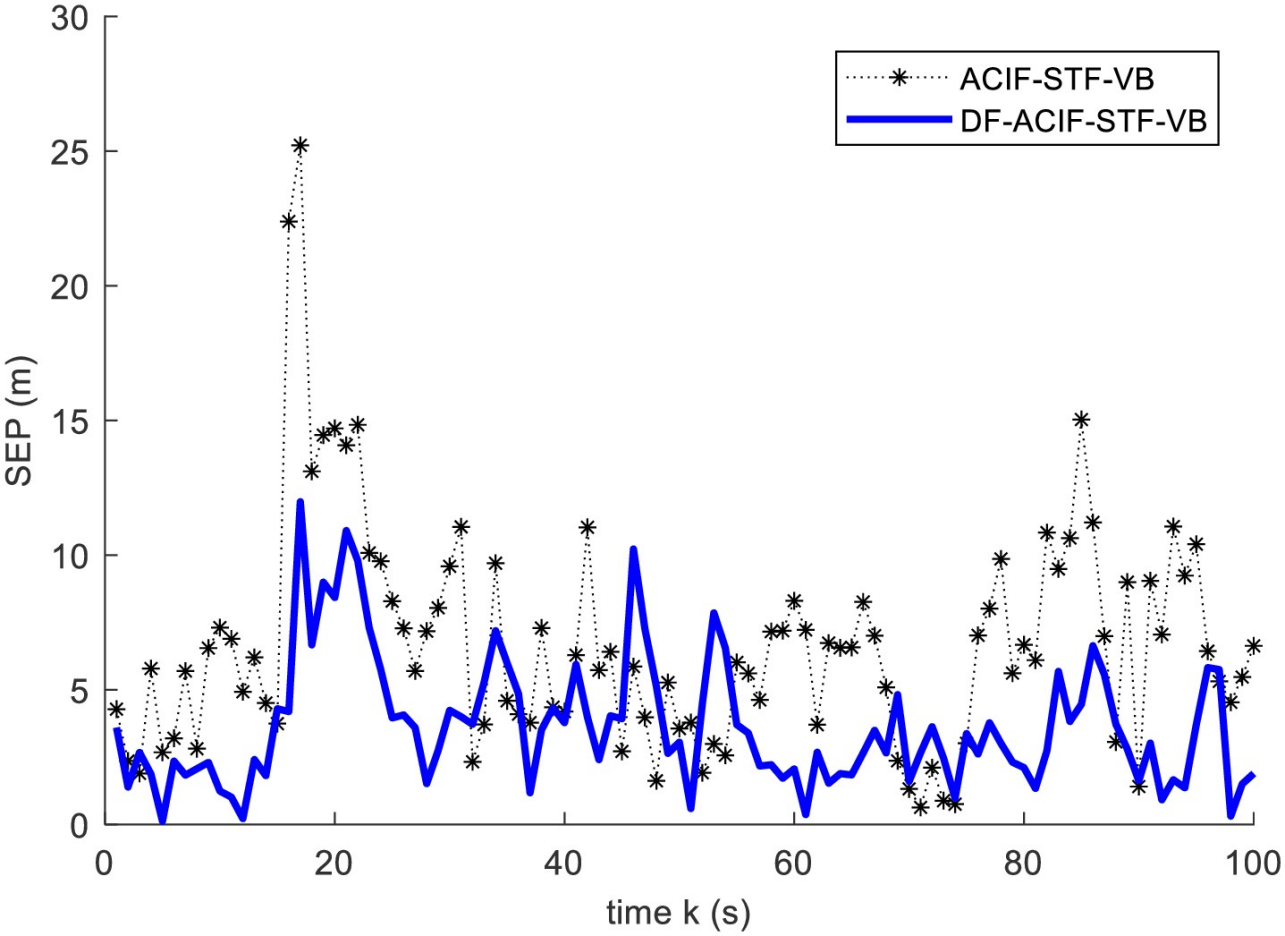
Square error curves of the position.

**Fig 5:**
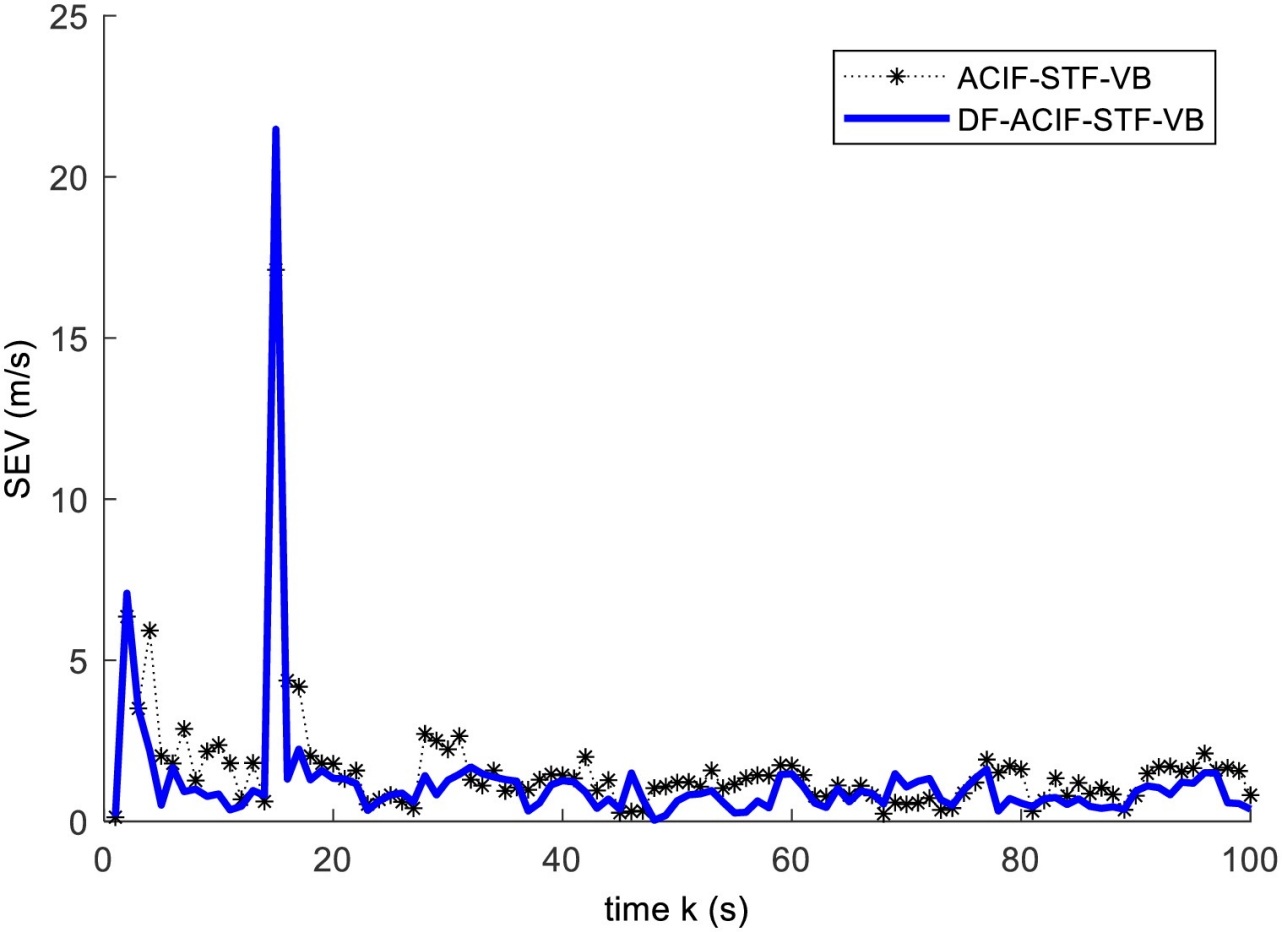
Square error curves of the velocity.

In this example, we compare the estimation accuracy of DF-ACIF-STF-VB and ACIF-STF-VB. Then, the results are given by Figs 4 and 5 and Table 2.

**Table 2:** Mean square error of two algorithms.

Algorithm	ACIF-STF-VB	DF-ACIF-STF-VB
MSEP (m)	6.6963	3.6528
MSEV (m/s)	1.5851	1.1956

From Figs 4 and 5, it is obvious that DF-ACIF-STF-VB has better estimation accuracy than ACIF-STF-VB. In addition, Table 2 also shows that mean square error of the former is lower than that of the latter. Specifically, the estimation accuracy of MSEP and MSEV of DF-ACIF-STF-VB increased by 45.45% and 24.57% respectively. These results are consistent with the general conclusion of multisensor fusion theory, this is, the fusion results should be superior to the single sensor results. All these results verify the effectiveness of the decentralized fusion algorithm. In other words, the calculation method of the global factor is effective.

## Conclusions

This article investigates the problem of multisensor nonlinear fusion with sudden change of state and unknown variance of measurement noise. In order to deal with these uncertainties, strong tracking filter (STF) and variational Bayesian (VB) technology are adopted in cubature information filter (CIF), a novel adaptive CIF is proposed, which is called ACIF-STF-VB. Compared with existing method in [15], ACIF-STF-VB does not require the evaluation of Jacobians and solves the interdependent problem on the computation of some parameters. Subsequently, proposed algorithm is easy to extend for multisensor state estimation in decentralized fusion framework with feedback. We define a parameter named fading vector, which is convenient to evaluate global fading factor and reduce the computational burden of fusion center. The experimental results demonstrate the effectiveness of the proposed algorithms. It will be an interesting future research topic to consider the fusion system with correlated noises.

## Supporting information

S1 Fig Information filter for decentralized fusion. (TIF)

S2 Fig Square error curves of the position. (TIF)

S3 Fig Square error curves of the velocity. (TIF)

S4 Fig Square error curves of the position. (TIF)

S5 Fig Square error curves of the velocity. (TIF)

S1 Table Mean square error of two algorithms. (PDF)

S2 Table Mean square error of two algorithms. (PDF)
