# Supplementary figures and images for: Multisensor decentralized nonlinear fusion using adaptive cubature information filter
Source: PLoS One. 2020 Nov 5;15(11):e0241517. doi: 10.1371/journal.pone.0241517 (PMC7643980; doi:10.1371/journal.pone.0241517)

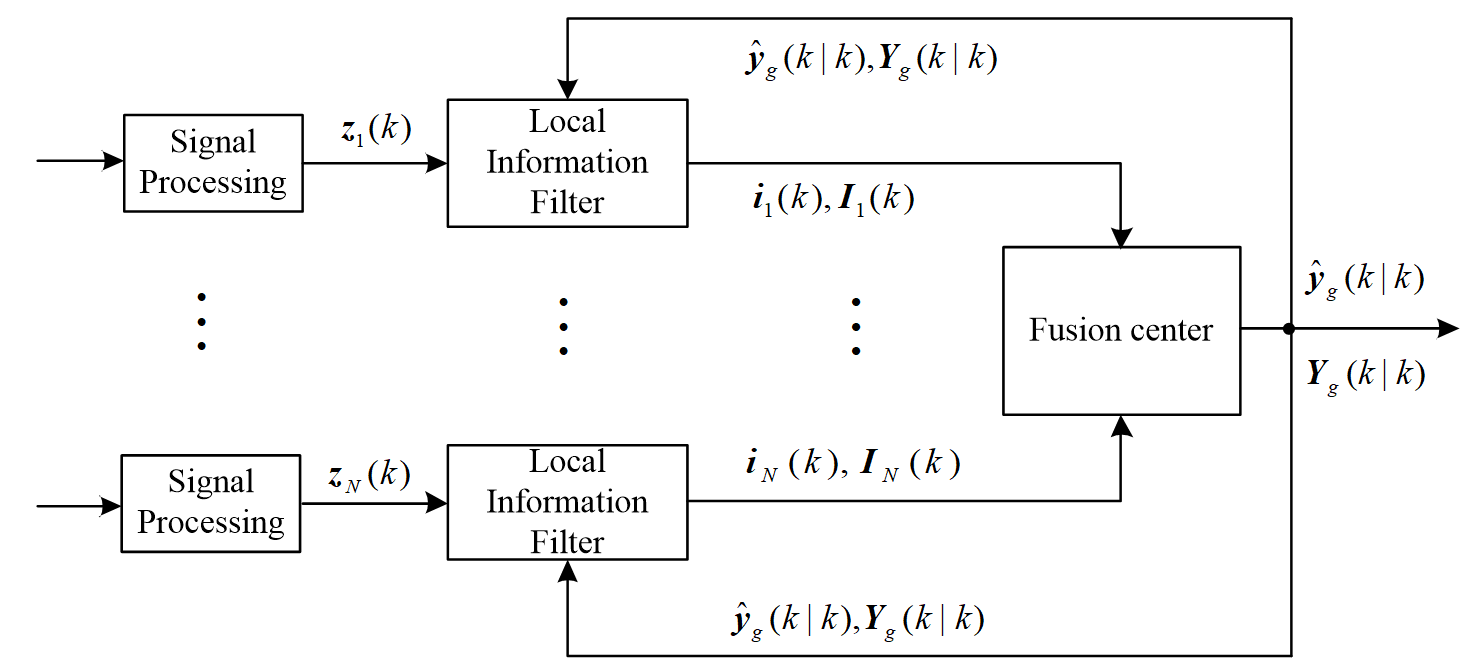

Supplement: S1 Fig — (TIF) [file pone.0241517.s001.tif]

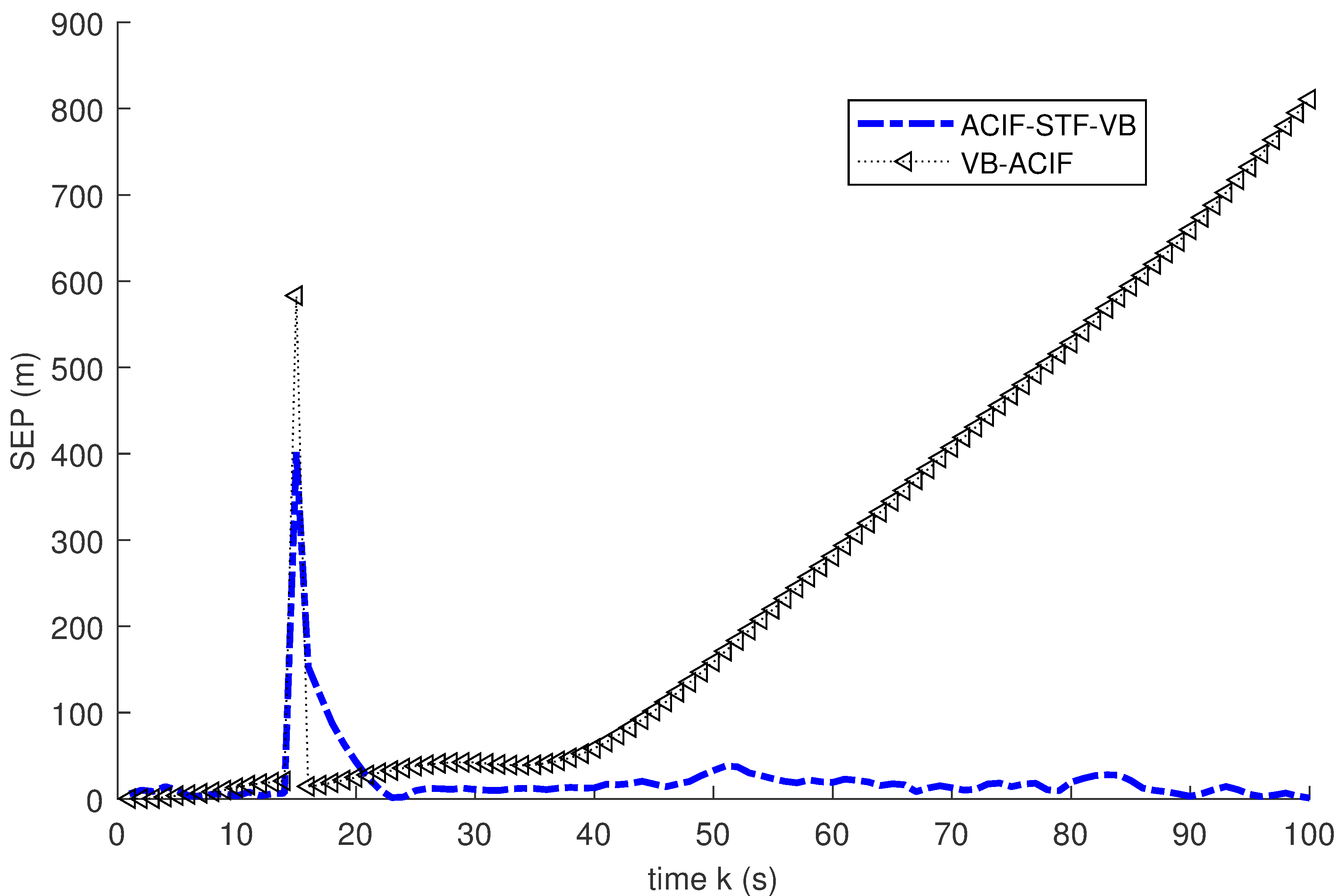

Supplement: S2 Fig — (TIF) [file pone.0241517.s002.tif]

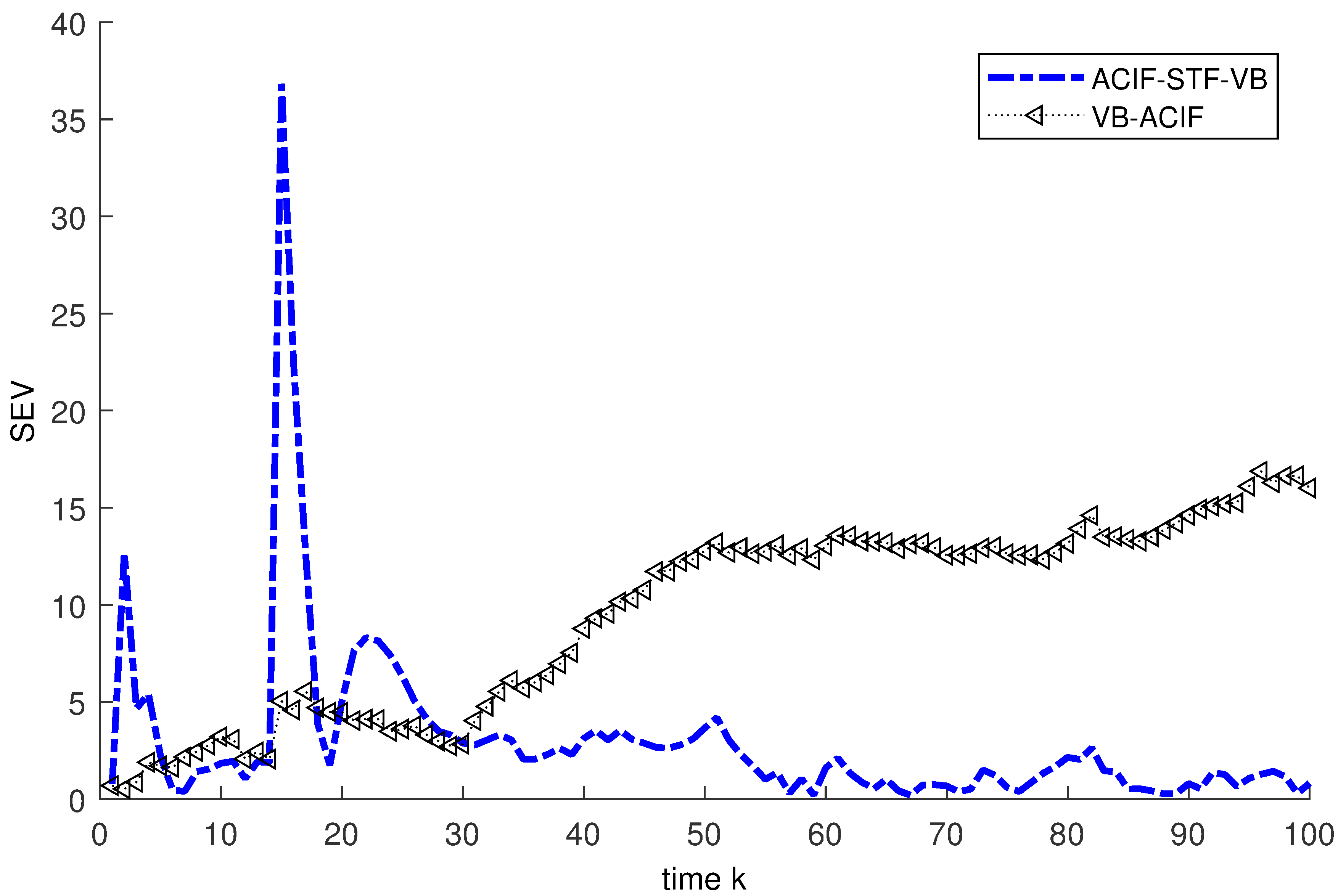

Supplement: S3 Fig — (TIF) [file pone.0241517.s003.tif]

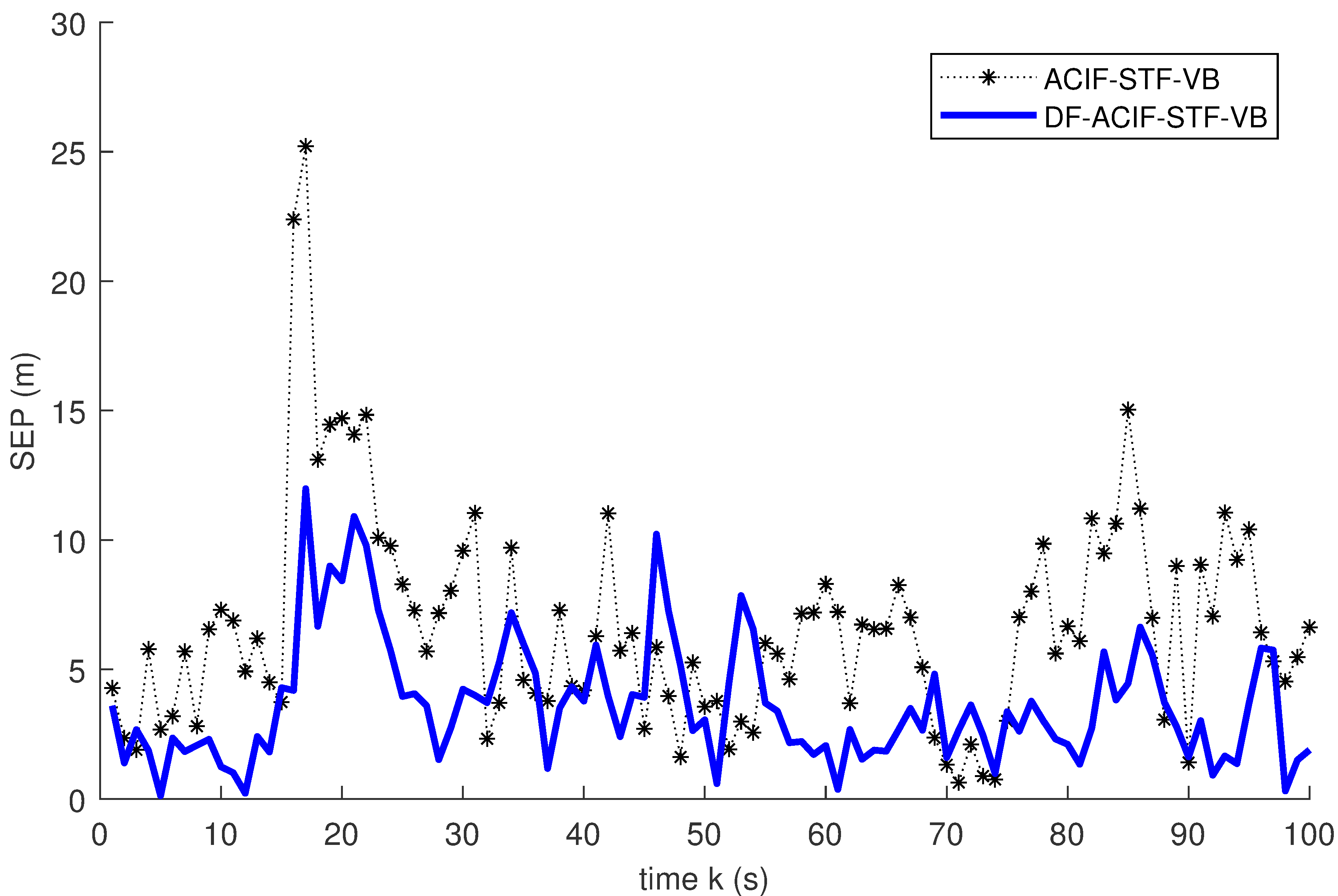

Supplement: S4 Fig — (TIF) [file pone.0241517.s004.tif]

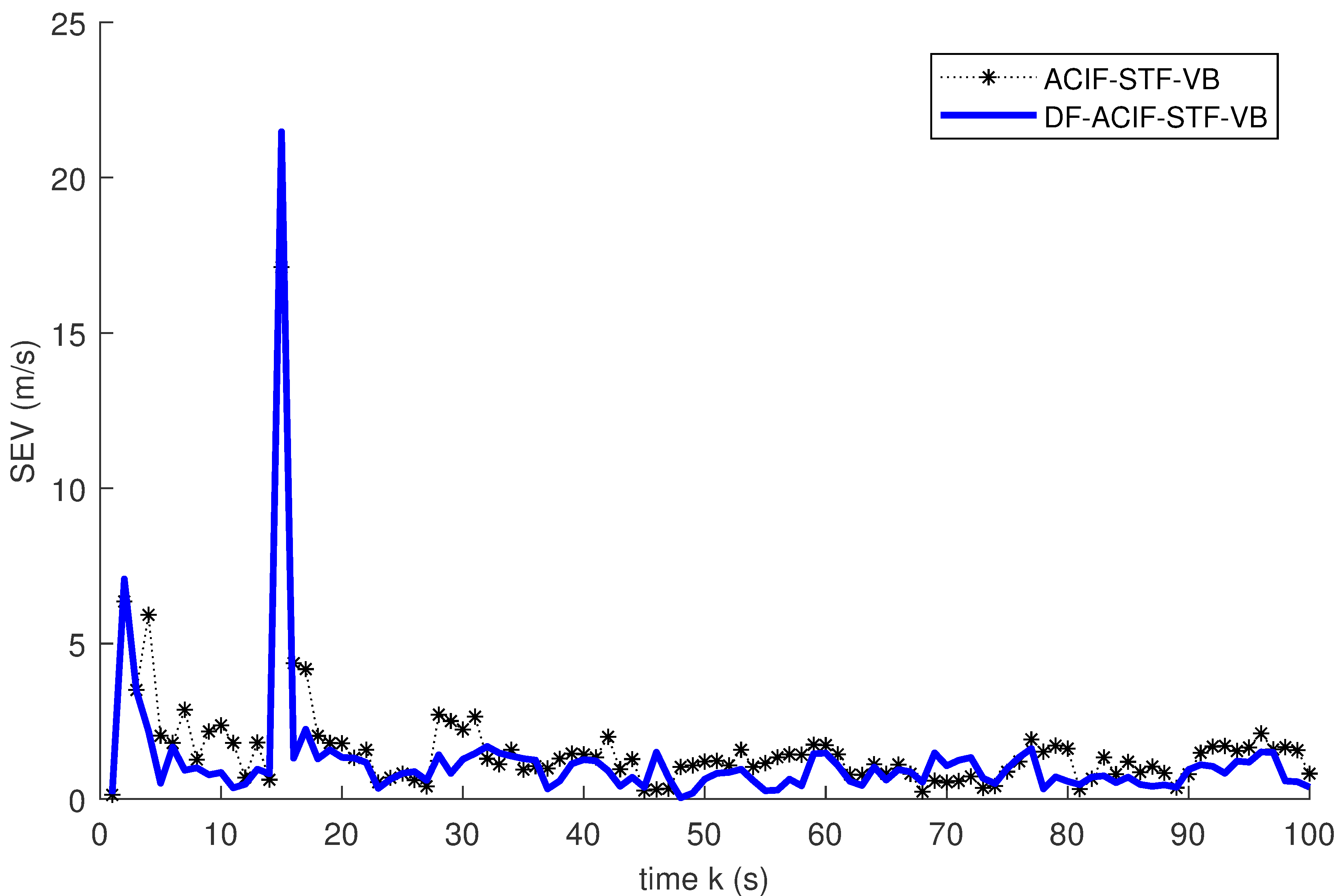

Supplement: S5 Fig — (TIF) [file pone.0241517.s005.tif]
