# Supplementary material for: Multisensor decentralized nonlinear fusion using adaptive cubature information filter
Source: PLoS One. 2020 Nov 5;15(11):e0241517. doi: 10.1371/journal.pone.0241517 (PMC7643980; doi:10.1371/journal.pone.0241517)
Supplement: S1 Table — (PDF) [file pone.0241517.s006.pdf]

**Algorithm**	**ACIF-STF-VB**	**VB-ACIF**
MSEP (m)	21.8446	266.3492
MSEV (m/s）	2.9034	9.4795
